# Supporting Rural Veterans With Vision Loss Using a Low-Tech Tool Kit: The First Step

**DOI:** 10.1093/geroni/igaf122.834

**Published:** 2025-12-31

**Authors:** Chiung-Ju Liu, Kimberly Findley, Consuelo Kreider, Hongwu Wang

**Affiliations:** University of Florida College of Public Health and Health Professions, Gainesville, Florida, United States; Malcom Randall VA Medical Center, Gainesville, Florida, United States; University of Florida College of Public Health and Health Professions, Gainesville, Florida, United States; University of Florida College of Public Health and Health Professions, Gainesville, Florida, United States

## Abstract

Age-related vision loss can significantly impact older people’s daily activities, leading to care dependency. Access to vision care services is challenging for rural older Veterans with vision loss due to long travel distances and limited capacity to drive. Low-tech solutions to enhance activity participation could be taught by VA generalist clinicians who are not vision specialists. For example, applying tactile cues to help operate a microwave. As an initial step of an implementation study, this survey aimed to understand 1) VA generalist clinicians’ awareness of older Veterans’ vision concerns and 2) their preferences for a tool kit that offers low-tech solutions to address these concerns. Community-based VA clinicians in the Northern Florida/South Georgia Veterans Health System were invited to complete the survey, with 39 respondents representing seven health professions. Descriptive analysis showed that 92% of respondents had served rural Veterans in the past three months, and only 59% routinely reviewed medical records for vision-related concerns. The top identified concerns were difficulty reading, driving, and falls. Respondents mostly referred the Veterans to vision care services (67%). Additionally, respondents’ top-ranked features for the low-tech tool kit as “very important” were: having simple instructions with clear illustrations (77%), kit items that are intuitive to use (64%), and items that can be easily replaced (64%). Survey results suggested a need to increase VA generalist clinicians’ awareness of activity challenges faced by rural older Veterans with vision loss. Results also guided the development of a low-tech tool kit to meet the clinicians’ preferences.

